# Precision pig feeding: a breakthrough toward sustainability

**DOI:** 10.1093/af/vfz006

**Published:** 2019-03-30

**Authors:** Candido Pomar, Aline Remus

**Affiliations:** Sherbrooke Research and Development Centre, Agriculture and Agri-Food Canada, Sherbrooke, QC, Canada

**Keywords:** modern livestock production, nutrient utilization efficiency, precision feeding, precision livestock farming, swine

**Implications**

• Utilization of precision feeding techniques in growing pig operations can significantly reduce production costs (>8%), protein and phosphorous intake (25%) and excretion (40%), and greenhouse gases emissions (6%) by increasing individual nutrient efficiency.
• Precision feeding allows real-time off-farm monitoring and intelligent management of feeds and animals for improved economic efficiency, significant reduction of labor requirements, and early identification of animal environmental and health stressors thereby, reducing use of antibiotics.
• Precision feeding is a major breakthrough in pig nutrition and one of the most promising avenues to promote high-quality and safe pork, high animal welfare, and minimal impact on the environment.

## Introduction

Swine production systems have dramatically changed in the last three decades. Today main challenges for the hog industry are to maximize feed efficiency while minimizing production costs and environmental impacts. With regard to environmental impacts, the issue lies mainly with nitrogen and phosphorus excretion which are reaching alarmingly high levels in most intensive pig production areas (Strid Eriksson et al., 2005; Garcia-Launay et al., 2014). The high relevance of environmental load has forced swine producers and nutritionists around the world to reassess the nutritional and feeding programs in use. Excretion of nutrients can be reduced by providing an individual animal with its required dietary levels. This practice also improves nutrient efficiency and reduces production costs (Pomar et al., 2015; Andretta et al., 2016).

Conventionally, factorial methods (NRC, 2012) are used to estimate nutrient requirements for pigs fed in large groups that receive the same feed for extended periods throughout their production cycle. In growing-finishing pigs, for example, their appetite increases faster than nutrient requirements and therefore, the optimal concentration of nutrients in the diet decreases as the pig gets older. These pigs are often fed with three feeds in three distinct feeding phases. The number of feeding phases can be increased to avoid oversupplying pigs with nutrients. Preferably, diets should be adjusted daily to account for the nutritional requirements of pigs more accurately, and therefore improve the efficiency of nutrient utilization. However, increasing the number of diets complicates feed management and increases production costs.

Precision livestock farming is an innovative production system approach that can be defined as the management of livestock using the principles and technologies of process engineering (Wathes et al., 2008). Precision animal nutrition or precision feeding is part of the precision livestock farming approach and involves the use of feeding techniques that allow the proper amount of feed with the suitable composition to be supplied in a timely manner to a group of animals (Parsons et al., 2007; Cangar et al., 2008; Pomar et al., 2014) or to individual animals (Andretta et al., 2014; Andretta et al., 2016) to enhance farm profitability, efficiency, and sustainability (Hauschild et al., 2012; Pomar and Pomar, 2012; Pomar et al., 2017). In this production system, the interanimal variability is taken into account by feeding pigs with diets tailored daily to their individual requirements (Pomar et al., 2009; Hauschild et al., 2012; Andretta et al., 2014).

The practical application of precision feeding, especially individual precision feeding, can have great impact on livestock sustainability. Precision feeding is a promising feeding technique to reduce the environmental footprint of pig production systems (Gerber et al., 2013). Precision feeding offers immediate and tangible benefits to the pork producer given that feeding pigs individually with daily tailored diets reduces lysine intake by more than 25%, feeding costs by more than 8%, nitrogen and phosphorus excretion by nearly 40% (Andretta et al., 2014; Andretta et al., 2016), and greenhouse gases emission by 6% (Andretta et al., 2018). Still, the actual on-farm application of precision feeding requires better understanding of variability among individual animals in terms of their physiological, behavioral, and production responses. Advanced scientific knowledge in animal sciences should be integrated with information and communication technologies for the development of precision feeding.

## Improving Nutrient Efficiency Reduces the Environmental Impact of Pig Production

Farm animals are raised to produce commodities such as food (i.e., meat, dairy products), fiber, and labor. The energy and nutrient losses associated with the conversion of feed nutrients to animal products increase production costs and the environmental load (i.e., nitrogen, phosphorus, trace minerals, carbon, and methane). Feed costs may represent between 60% and 70% of the overall production costs in various species such as pigs, poultry, and cattle. However, the efficiency by which domestic animals transform nutrients in feed into animal products is generally low. For instance, protein (i.e., nitrogen), which is among the most limiting and expensive nutrient in livestock feeds, is retained by growing pigs with efficiency normally ranging from 15% (Flachowsky and Kamphues, 2012) to 33% (Dourmad et al., 1999). Similar figures are found for converting dietary protein into meat protein in beef cattle and broilers where the efficiency ranges from 10% to 20%, and from 30% to 40%, respectively (Flachowsky and Kamphues, 2012). The protein in the feed that is not incorporated into animal products is excreted and can result in environmental problems such as nitrate pollution of aquifers, and pollution of surface water with problems such as algal bloom. Improving nutrient efficiency is essential because of the challenges associated with the expected increase in the human population, limited arable land, and the environmental problems that are frequently associated with farm animal production (Niemann et al., 2011).

There are various sources of nutrient inefficiency within the animal. First, portions of the ingested nutrients are used for basal metabolic processes involving degradation (catabolism) and synthesis (anabolism), or are lost in the digestive tract through desquamation and endogenous secretions. These losses are generally referred to as maintenance losses. Nutrients are also lost during the production of animal products (e.g., body lean). In growing animals, the losses associated with utilization of the first-limiting amino acid for body protein deposition can largely be attributed to its inevitable catabolism (Mohn et al., 2000). These inevitable amino acid losses should be differentiated from other metabolic losses related to the preferential amino acid catabolism, which results from the catabolism of amino acids given in excess, from the excretion of chemically unavailable absorbed amino acids (e.g., heat-damaged proteins) (van Barneveld et al., 1994), and from the use of amino acids for the synthesis of nonprotein body compounds (Moughan, 1989). In growing animals fed with cereal-based diets, the sum of the undigested nitrogen and the losses associated with digestion, maintenance functions, and body protein deposition may represent 33% of the total ingested nitrogen. Similar values are obtained for dietary phosphorus (Dourmad et al., 1999). These sources of nutrient inefficiency are difficult to minimize because they occur during digestion and metabolic processes.

**Figure 1.**
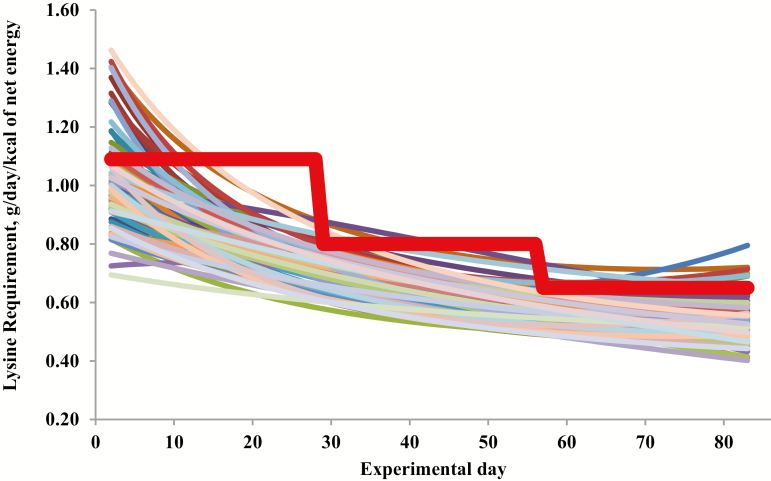
Estimated standardized ileal digestible lysine requirements of individual pigs (thin colored lines) and minimal standardized ileal digestible lysine levels to be provided to pigs fed in a conventional group three-phase feeding system (bold red line) without affecting body weight gain according to Hauschild et al. (2010).

Besides the inevitable nutrient losses associated with digestion and metabolism, growing pigs may receive more nutrients than they need and all nutrients given in excess are excreted and contribute to the overall nutrient inefficiency. Pigs are raised and fed in groups, usually with the same feed which is provided to all animals of the group during a given period of time. However, nutrient needs largely vary among animals in a population (Figure 1) and these needs evolve over time following individual patterns (Hauschild et al., 2010). Therefore, two important sources of variation must be controlled to improve animal production efficiency. These sources of variation are the variation between animals within the group receiving the same feed, and the changes in individual or group nutrient requirements over time. Given that for most nutrients underfed animals will exhibit reduced performance, whereas the overfed ones exhibit near optimal performance, nutrients have to be provided to satisfy the requirements of the most demanding animals in the group to obtain optimal production performance (i.e., growth) (Pomar et al., 2003; Brossard et al., 2009; Hauschild et al., 2010). In this situation, almost all animals receive more nutrients than they need. Providing animals with a high level of nutrients to maximize herd performance is common practice in commercial livestock operations, even though maximum growth does not ensure maximum economic efficiency (Hauschild et al., 2010; Niemi et al., 2010). Furthermore, to account for the variability between animals, feed ingredient composition, and other uncontrolled and unknown factors (e.g., environment, health), nutritionists include safety margins when formulating diets for maximum population responses. The need of these safety margins can be seen as an admission of our inability to precisely estimate the nutrient requirements of groups of animals (Patience, 1996).

Nitrogen and phosphorus conversion efficiency can vary between 10% and 40% depending on the animal, diet, and farm management. The conversion of dietary nitrogen into animal protein is on average more efficient in monogastric animals than in polygastric animals (Flachowsky and Kamphues, 2012). Taking into account the efficiency and the number of animals, the largest nitrogen manure producers are cattle, sheep, and pigs contributing 60%, 12%, and 6%, respectively, of total manure nitrogen (Oenema and Tamminga, 2005). It is important to note that these differences in nitrogen efficiency can be as large within a type of animal as between production systems. Control of the management of animals and animal feed are the most important factors to reduce N excretion (Oenema and Tamminga, 2005).

Production efficiency (especially nutrient efficiency) and environmental impacts are strongly correlated. In fact, dietary nutrients that are not retained by the animal or in animal products are excreted via the urine and feces as well as some greenhouse gases (e.g., methane). Reducing nutrient intake without limiting animal performance is therefore the most efficient way to reduce nutrient losses. For example, for each percent unit of reduction of protein intake, nitrogen excretion can be decreased by 1.5%. Besides the reduction in protein intake and excretion, feeding costs are also reduced (Andretta et al., 2016). Fortunately, precision feeding significantly improves nutrient efficiency by controlling the two identified sources of evitable nutrient losses, that is, those related to the between-animal variation and those related to the individual evolution of nutrient requirements. Furthermore, precision feeding needs to include much lower safety margins than conventional feeding.

## Implementation of Precision Feeding

Precision feeding concerns the use of feeding techniques that provide animals with diets tailored according to the production objectives (i.e., maximum or controlled production rates), including environmental impact and animal welfare issues. Precision feeding is presented in this document as the practice of feeding individual animals while accounting for the changes in nutrient requirements that occur over time and for the variation in nutrient requirements that exists among animals. Accurate determination of available nutrients in feed ingredients, precise diet formulation, and determination of the nutrient requirements of individual animals or group of animals should be included in the development of precision feeding systems (Van Kempen and Simmins, 1997; Pomar et al., 2009). Implementation of precision feeding systems in commercial farms requires the integration of three types of activities: 1) automatic collection of data, 2) data processing according to the established control strategy, and 3) actions concerning control of the system (Aerts et al., 2003; Banhazi et al., 2012b). Application of precision feeding at the individual level is only possible where measurements, data processing, and control actions can be applied to the individual animal (Wathes et al., 2008).

### Data collection

Measurements on the animal, the feeds, and the environment are essential for precision feeding and these parameters have to be measured directly and frequently (if possible, continuously). In fact, we cannot manage and control a system without appropriate measurements. Essential measurements for precision feeding in growing pig operations include feed intake and body weight. The availability and the rapid development of new devices and emerging sensor technologies offer great potential for other measurements (e.g., body composition, physical activity, interactions among animals) that will allow more precise estimation of requirements and real-time animal monitoring.

### Data processing

Collected data has to be processed according to the farm production objectives. There are several potential control strategies available for the application of precision feeding in swine operations. In animals offered feed ad libitum, the only way to control nutrient intake is by varying the composition of the feed to be served. In this situation, both the between-animal variation and the time-dependent nutrient requirement variation can be controlled. In contrast, in animals that are offered feed restrictively the amount and the composition of the feed can be easily controlled.

Mathematical modeling is a methodology used to understand and to quantify complex biological phenomena involved in animal production and it is the basis for data processing in precision feeding systems. Mathematical models developed for precision feeding, however, have to be designed to operate in real-time using real-time system measurements. Therefore, they are structurally different from traditional nutrition models, which are developed to work in a retrospective manner and to simulate known production situations. The first mathematical model developed to estimate in real-time individual pig nutrient requirements was proposed by Hauschild et al. (2012). The required daily concentration of lysine is estimated in this model using individual feed intake and body weight information. Using these data, an empirical model component estimates the expected body weight, feed intake, and weight gain for the next day, whereas a mechanistic model component uses these three estimated variables to calculate with a factorial method the optimal concentration of lysine that should be offered that day to each pig in the herd to meet its requirements. Other amino acids and nutrient requirements are assumed proportional to the lysine requirements.

### Control of the system

**Figure 2.**
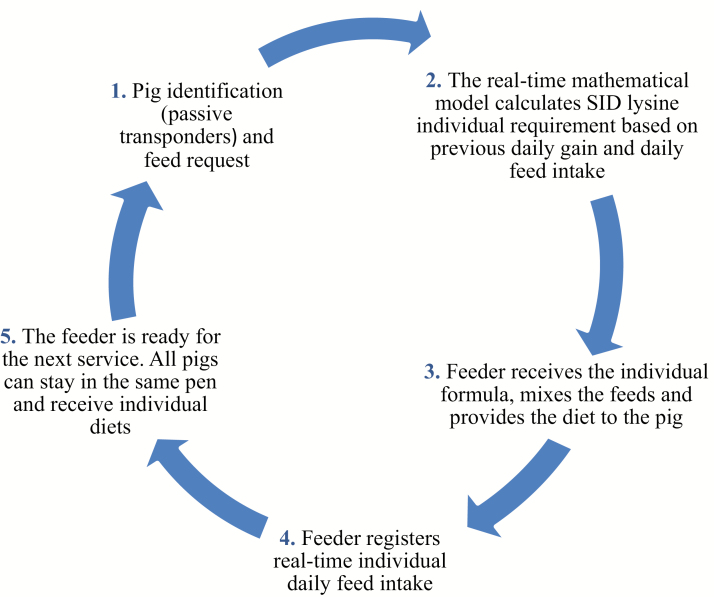
Scheme of the automatic precision feeding system operation using individual pig actual daily gain and daily feed intake to predict individual standardized ileal digestible (SID) lysine requirements.

The information collected and processed is used to control the production system. In the context of precision feeding, automatic precision feeders are used to provide individual pigs with the right amount and composition of the feed at a given time. Plastic button tags inserted in the ear contain passive transponders (RFID) that are used for pig identification (Figure 2). At least two feeds (named A and B) are needed for precision feeding. These two feeds should be formulated on the basis of net energy, standardized ileal digestible amino acids and other essential nutrients. Feed A (high nutrient density feed) is formulated for the most demanding pigs at the beginning of the growing period, whereas feed B (low nutrient density feed) is formulated for the less demanding pigs at the end of the finishing period. Blending feeds A and B at different proportions allows the feeders to provide individual pigs with the right feed. The feeders consist of a single space trough in which precision Archimedes’ screw conveyors deliver and blend simultaneously volumetric amounts of two feeds contained in independent feed containers. The feeder identifies each pig when their head is introduced into the feeder and the feeds are blended and delivered upon the animal’s request (according to the estimated optimal lysine concentration). A serving is composed of the amount of feed delivered upon each effective serving request. A time lag is imposed to ensure that pigs eat each serving before requesting a new serving. Serving size is progressively increased and ranges between 15 and 25 g (Pomar et al., 2011). A meal includes all the servings delivered during each feeder visit. Pigs tend to leave the feeder trough empty or leave very small amounts of feed after each visit, thus ensuring that each pig receives the assigned amount of blended feed. Feed density needs to be measured weekly and this information should be used to convert feed volumes to feed weights.

**Figure 3.**
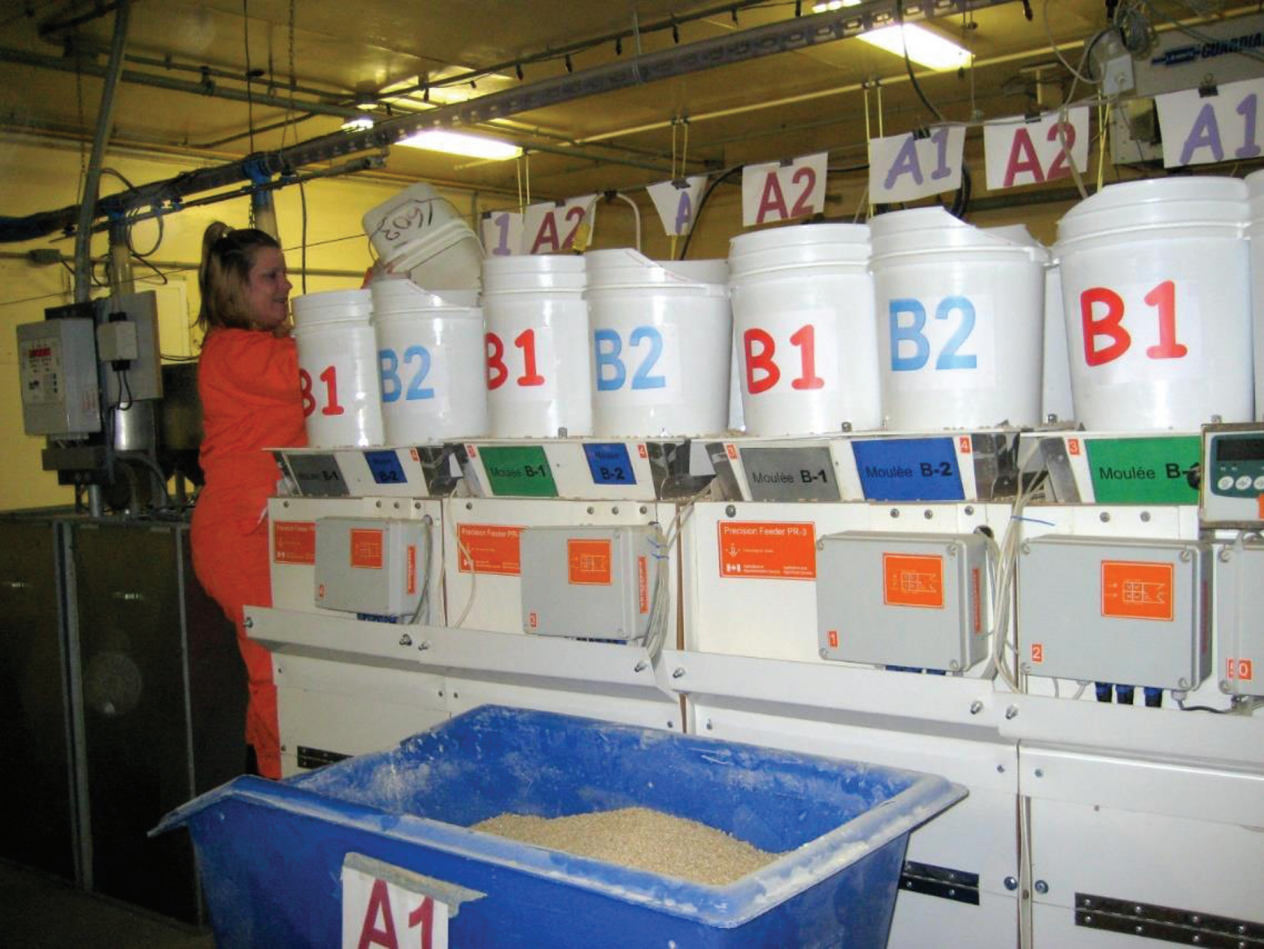
Calibrating the precision feeding mathematical model (Cloutier et al., 2015) using four feeds in each feeder. Feeds A1 (130% of lysine requirements) and A2 (70% of lysine requirements) are formulated to meet the pig’s highest lysine requirements, and B1 (130% of lysine requirements) and B2 (70% of lysine requirements) are formulated to meet the pig’s lowest lysine requirements.

**Figure 4.**
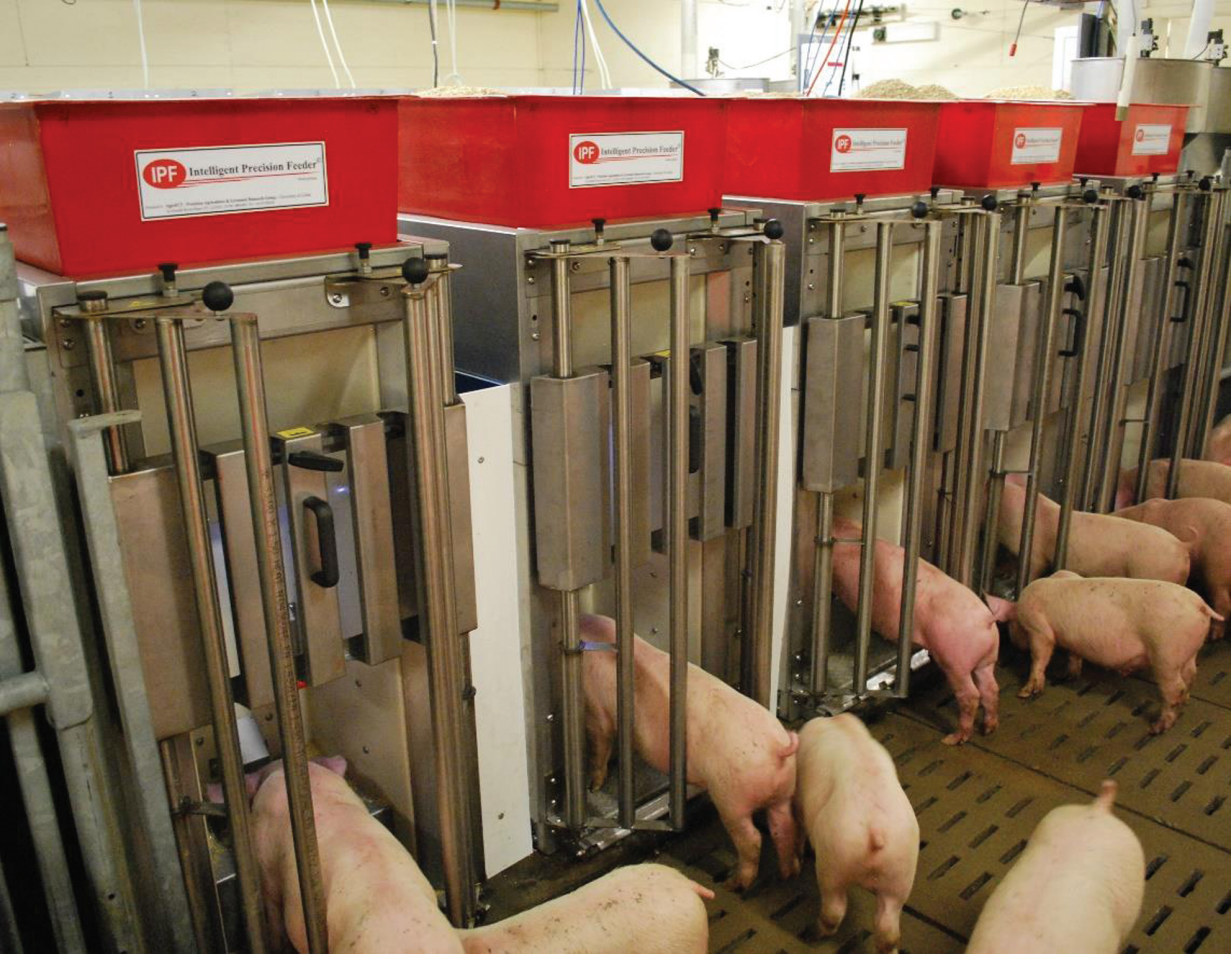
Individual feeders allow one pig at time to request feed. Each animal is identified by the ear plastic button tags containing passive transponders, which provide pigs with diets that are tailored daily to individual requirements of each pig.

A real-time modeling-control approach was used by Pomar et al. (2014) to control the time-dependent variation of group-housed pigs offered feed ad libitum. Comparing the traditional three-phase feeding system to the daily-phase feeding system, these authors concluded that protein intake could be reduced by 7% while nitrogen excretion was reduced by 12%. Controlling the time-dependent and the between-animal variation can further help the reduction of nutrient intake and excretion. The modeling approach proposed by Hauschild et al. (2012) was used to estimate real-time nutrient requirements in individual pigs, was calibrated in two animal trials (Zhang et al., 2012; Cloutier et al., 2015; Figure 3), and the overall approach of estimating real-time amino acid requirements was challenged in two validation trials (Andretta et al., 2014; Andretta et al., 2016; Figure 4). The latter authors showed that daily adjustment of the diet resulted in a 27% reduction in total lysine supply, without detrimental effects on growth. This additional 20% reduction in lysine intake in relation to group-fed pigs could be obtained by feeding the animals individually and thus controlling simultaneously the time-dependent and the between-animal variation. Although feed cost reduction depends to a great extent on feed prices, it is expected that feed cost can be reduced by 1% to 3% when only controlling the time-dependent variation while an 8% to 10% reduction can be obtained when controlling both sources of variation. Nitrogen excretion was reduced by nearly 30% when pigs were fed with daily tailored diets.

## Future Perspectives

To further develop precision feeding systems, it is necessary to improve our actual understanding of several animal metabolic processes. Precision feeding is still based on mathematical models and nutritional concepts developed for average population responses. When feeding individual pigs with daily tailored diets, these traditional nutritional concepts are not accurate and even sometimes incorrect (Remus et al., 2017; Remus, 2018). It is necessary to distinguish the nutritional requirements of a population from those of an individual. Individual pigs are able to modulate growth and the composition of growth according to the level of available amino acids (Remus, 2018). Also, pigs can respond differently to the same amount of ingested amino acids, due to differences in the efficiency of amino acid utilization. These aspects are not considered in current nutritional models, which assume that the efficiency by which animals use the available amino acids is constant. Similarly, the amino acid composition of whole body protein is assumed to be constant, whereas it has been shown to vary. Similar results have been found for the efficiency of calcium and phosphorus utilization (Gonzalo et al., 2018). Understanding the metabolic processes responsible for the observed variation between individual animals in their ability to use dietary nutrients is challenging nutritionists and modelers but is required to further improve the efficiency of livestock production. Advances in precision feeding rely on the development of sound nutritional concepts and comprehensive biological models to more precisely estimate individual real-time nutrient requirements. The new understanding of individual metabolism and nutrition will allow animal science to move forward, opening up new opportunities for individualized nutrition. Continuous and automatic monitoring of animals and farm resources will support production decisions at the farm level, the early detection of diseases and thus, decrease the use of antibiotics and avoid the spread of infectious diseases. This will ultimately enhance farm profitability, efficiency, and sustainability of the overall production system (Banhazi et al., 2012a).

The mathematical model developed to estimate daily lysine requirements in individual growing-finishing pigs (Hauschild et al., 2012; Zhang et al., 2012; Cloutier et al., 2015) is being updated to account for the variation in amino acid efficiency and the requirements of amino acids other than lysine (Remus et al., 2017; Ghimire et al., 2016). Further developments will also include new knowledge concerning the genetic capability of pigs to efficiently use nutrients and integrate in the daily estimation of individual pigs’ optimal nutrient requirements, the interaction between feeding patterns, diet composition, and the digestive and metabolic dynamic availability of dietary nutrients. These model improvements will further reduce the environmental footprint of the swine industry with estimated reductions of feed cost of more than 12%, nitrogen and phosphorus excretion of more than 60%, and greenhouse gas emissions of over 12%.

## Conclusion

Precision feeding is a major breakthrough in pig nutrition and one of the most promising avenues to promote high-quality and safe pork with the lowest environmental impact (60% less nutrient excretion) and high animal welfare standards. Fewer pollutants would mean improved population wellness and health as well as reduced odors, harmful waste, and the risks of water, air (e.g., ammonia and greenhouse gas emissions), and soil pollution. Managing feeds and animals by means of advanced computerized technologies make it possible to identify diseases early and apply individual treatments precisely to improve herd performance, reduce antibiotic use, and contribute to improved public safety.

## About the Authors

**Dr Candido Pomar** is a senior research scientist with Agriculture and Agri-Food Canada at Sherbrooke Research and Development Centre, Quebec, since 1991. He is adjunct professor at Laval and Sherbrooke universities in Québec, and UNESP university in Sao Paolo, Brazil. He obtained his Engineering degree at Madrid Polytechnic University (Spain) and his Master and PhD degrees at Laval University, after working at Michigan University and the USDA ARS US Meat Animal Research Center (US MARC) in Clay Center, Nebraska. Dr Pomar is heading a research program in swine nutrition, mathematical modeling, and carcass evaluation systems and is engaged in the development of innovative precision feeding and farming systems.

**Dr Aline Remus** is a postdoctoral researcher with expertise in amino acid metabolism and requirements in individual and group precision feeding systems. She obtained her BSc in Animal Science (major in nonruminant feeding and nutrition) at the Universidade Federal de Santa Maria (Brazil), her Master degree in Animal Science at the Estadual Paulista “Júlio de Mesquita Filho” University (Unesp; Brazil), and her PhD as dual-degree doctorate at Unesp and Université Laval (Québec, Canada).
